# A case of gastric cancer associated to situs inversus totalis

**DOI:** 10.1186/1757-1626-1-391

**Published:** 2008-12-12

**Authors:** El Bachir Benjelloun, Fatima Ezzahra Zahid, Abdelmalek Ousadden, Khalid Mazaz, Khalid Ait Taleb

**Affiliations:** 1 Department of general surgery, University hospital Hassan II, Fès, Morocco

## Abstract

The situs inversus is a rare congenital anomaly, which is a more or less complete inversion of the abdominal and thoracic organs. We report a case of 70 years old man, without pathological antecedents complaining about epigastric pains associated to haematemesis, and whose gastric endoscopy objectified a gastric tumor. The pulmonary x-ray and the abdominal computed tomography (CT) revealed the previously unrecognized situs inversus totalis. A subtotal gastrectomy was performed and patient had an uneventful postoperative course.

## Background

Situs inversus totalis is a congenital anomaly, occurring at an incidence of 1 in 10 000–50 000 live births [1]. It's defined as a complete mirror image of the thoracic and abdominal viscera, although this does not seem to affect normal health or life expectancy, and it is not considered to be premalignant. It is detected accidentally at the time of the radiological investigation. In fact, there is little information about the abdominal manifestations of situs anomalies in adult. We report a case of situs inversus totalis found in association with gastric cancer in an elderly man. We describe this case because of the rarity of this anomaly, and it is important in that its recognition may help avoid mishaps at surgery or other interventions, particularly in the emergency setting.

## Case presentation

**Figure 1:**
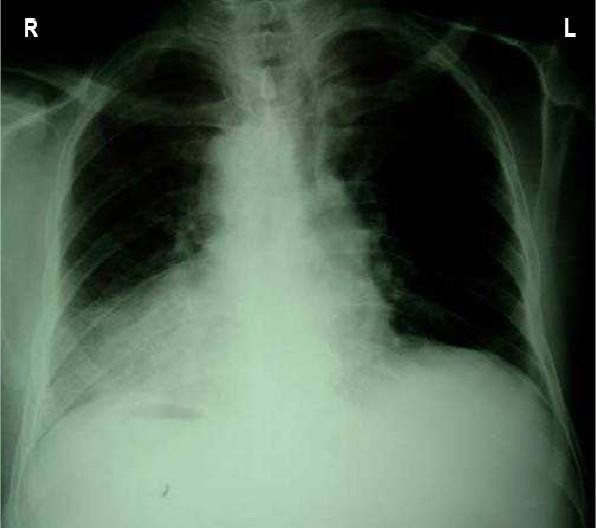
Chest X-ray demonstrating dextracardia.

A 70-year-old man was admitted to our hospital for treatment of gastric cancer. He had presented with a 7-month history of fatigue, intermittent abdominal pain, loss of appetite, vomiting and two episodes of haematemesis. He had lost 10 kg in weight during the last month, and felt he had no energy. The patient had no prior history of diabetes, hypertension, abdominal surgery or trauma. He had no cancer or weight loss. She didn't use any specific medication or drugs. The patient didn't smoke or drink alcohol. Physical examination revealed a temperature of 37°C, a pulse rate of 80 beat per minute (bpm), a blood pressure of 130/80 mm Hg. The patient was pale looking; the apex beat was in the right fifth intercostal space, midclavicular line. The heart sounds were normal. Nothing abnormal was found in the respiratory system. Abdominal examination revealed that the liver dullness was on the left side. There was a little tenderness in the epigastrium, but no mass was felt. On rectal examination the prostate was found to be moderately enlarged. The laboratory findings were: an hematocrit of 25%, an hemoglobin of 10 g/dl, a white blood cells of 8500/mm^3^, blood urea of 12 mg/dL, a creatinine level of 0,7 mg/dL. Dextrocardia was seen on the chest x-ray (Fig. 1), the electrocardiography findings were typical of congenital dextrocardia. Endoscopic examination objectified a tumor with ulceration in the antrum of the stomach. Histological examination of biopsied specimens resulted in a diagnosis of well differentiated adenocarcinoma.

**Figure 2:**
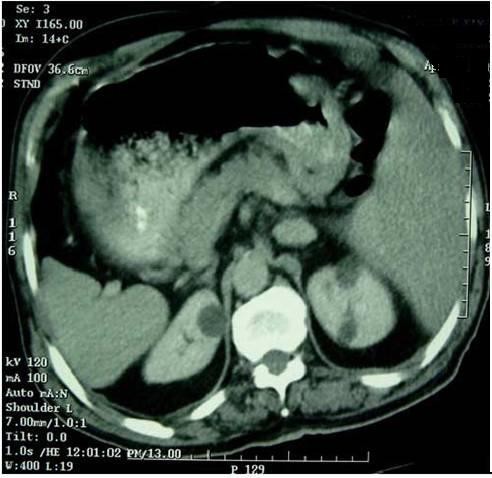
Computer tomogram of abdomen. Tumor thickened antrum gastric wall and situs inversus totalis.

Chest X-ray revealed a right sided heart (fig. 1). The abdominal CT showed Tumor thickened antrum gastric wall with situs inversus totalis (fig. 2).

**Figure 3:**
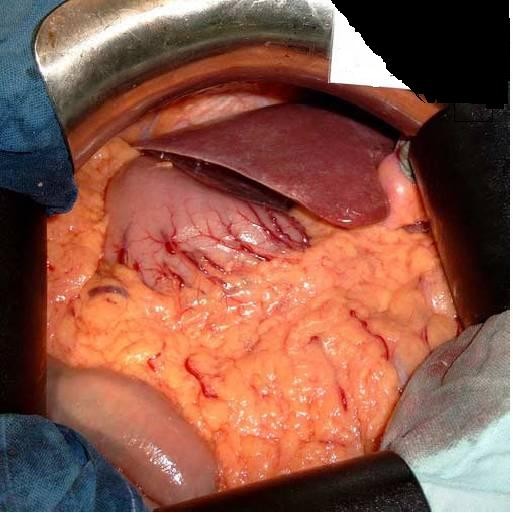
Laparotomy confirmed that the patient had situs inversus totalis.

**Figure 4:**
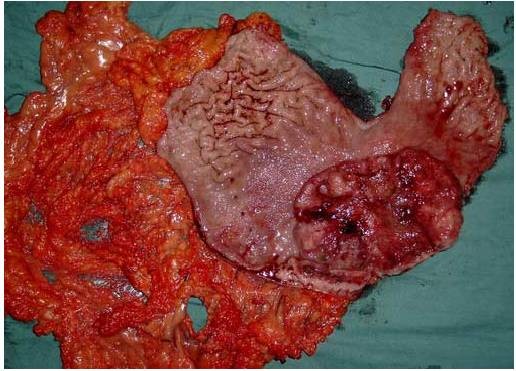
The open resected specimen with antrum tumor.

Based on the endoscopic and imaging findings we made a decision to perform a laparotomy. The abdomen was explored through a midline epigastric incision. There was complete transposition of the viscera (fig. 3), the stomach and spleen lying on the right side, and the gall-bladder, the larger lobe of the liver and the caecum and appendix on the left. On the posterior wall of the antrum of the stomach was a large carcinoma 6 cm/8 cm (Fig 4). There was no macroscopic serosal invasion or swelling of the lymph nodes. There was no evidence of glandular, hepatic, or peritoneal metastases. A subtotal gastrectomy Finsterer type was performed, with regional lymphadenectomy (D2), without splenectomy.

Histologic examination of the specimen showed a liberkunien middle differentiated adenocarcinoma, with serosal invasion and 3 lymph nodes metastasis (T3N+ according to international classification). The patient's postoperative course was uneventful. She was commenced on chemotherapy.

## Discussion

In 1600 the first known case of situs inversus in humans was reported by Fabricius [2]. The term "situs" refers to the position of the heart and viscera relative to midline. Situs solitus represents the normal position of the heart and abdominal viscera, with the cardiac apex, spleen, stomach, and aorta located on the left and the liver and inferior vena cava located on the right. Situs inversus totalis or situs inversus with dextrocardia indicates mirror-image location of the heart and viscera relative to situs solitus. Congenital heart disease occurs in 3%–5% of cases [3]. In contrast, situs inversus with levocardia is an extremely rare variant that is characterized by mirror-image location of the viscera relative to situs solitus and a left-sided cardiac apex. Nearly all affected individuals have congenital heart disease [4].

In 1936, Allen described a case of a prepyloric carcinoma of the stomach in a 30-year-old man with situs inversus and who three weeks after gastrectomy died [5]. There is no current evidence that situs inversus predisposes to gastric cancer [6,7].

The diagnosis of complete visceral transposition itself should not be currently difficult. However, if abdominal viscera alone are involved, the incorrect position of the diseased organ may be suspected. In our patient, thoracic and abdominal situs inversus was detected during the physical examination and routine medical evaluations on admission.

CT with multiplanar reconstruction (MPR) has become an established technique for the noninvasive imaging of abdominal vessels. With the availability of high resolution CT data sets, a wide variety of two-dimensional and three-dimensional reformations can now be acquired [7]. Because the chances of vascular anomalies, accompanying situs inversus are reported to be high, angiography is recommended if possible in such cases [8]. In our case, CT proved helpful in delineating the variations in the abdominal vessels, which enabled us to understand the regional anatomy.

The operation itself presented little difficulty. Some surgeons recommend reversing the positions of the operator and the assistant during surgery, but this do not seem to be critical. Needless to say, careful and cautious assessment of abnormalities by preoperative examinations is very important before any surgical procedure is performed, especially for laparoscopic procedures [9].

Subtotal gastrectomy with lymph node dissection (D2) is the preferred operation for malignant antrum cancer of the stomach due to the high incidences of multiple lesions of the stomach and involvement of the regional lymph nodes [10]. Chemotherapy is also essential if there is lymph node metastasis, as we recognised in our patient.

## Conclusion

Situs inversus presenting with gastric cancer is very rare. Preoperative information and good adaptation to the mirror image anatomy are essential for planning appropriate intervention.

## Consent

Written informed consent was obtained for publication of this case report and accompanying images. A copy of the written consent is available for review by the Editor-in-Chief of this journal."

## Competing interests

The authors declare that they have no competing interests.

## Authors' contributions

EBB is a surgeon who was drafting the manuscript and revising it critically for content. FZ is a surgeon who was involved in literature research. KM, KA were surgeons treating of the patient and were involved in revising the draft critically for content. OA is a surgeon was getting photographs and was involved in drafting    manuscript. All authors have given final approval of the revision to be published.  
